# Structural Basis of MERS‐CoV Receptor Interactions and Antibody Neutralisations

**DOI:** 10.1002/rmv.70113

**Published:** 2026-02-27

**Authors:** Edem Gavor, Yeu Khai Choong, Sunil Singh, Hariharan Sivaraman, Er Shi Yin, J. Sivaraman

**Affiliations:** ^1^ Department of Biological Sciences National University of Singapore Singapore Singapore

**Keywords:** antibodies, evolution, MERS‐CoV, receptors, SARS‐CoV, SARS‐CoV‐2

## Abstract

Increasing outbreaks of coronaviruses underscore the importance of antivirals and vaccines that can combat a wide range of coronaviruses. Neutralising antibodies (nAbs), along with vaccines and small‐molecule drugs, are among the most promising treatments and prevention options against coronaviruses. Here, we focus on Middle East Respiratory Syndrome coronavirus (MERS‐CoV) and discuss receptor usage and current progress in antibody research against MERS‐CoV infections. First detected in Saudi Arabia and Jordan in 2012, MERS‐CoV is a lethal zoonotic pathogen. MERS‐CoV infections have been reported by 27 countries between April 2012 till now, with 953 deaths (∼35% mortality) (5 new infections and 4 fatalities reported as of 1 October 2024). WHO identified MERS‐CoV as a high‐threat pathogen due to its severity, high mortality rate, and potential for epidemic or pandemic spread with recent outbreaks and deaths raising more concerns amidst the COVID‐19 pandemic. As of now, there is no antiviral drugs or vaccine against MERS‐CoV available. Here we provide a perspective on receptor usage, the risk of MERS‐CoV and other CoVs evolution on future pandemics, and the mechanisms of MERS‐CoV‐derived nAbs. We offer insight into how these antibodies cross‐react and cross‐neutralise by analysing available structures of spike glycoprotein–antibody complexes. This review provides an update and a basis for the development of antibodies and vaccines for MERS‐CoV, and possibly for the designing of next‐generation pan‐coronavirus vaccines and antivirals.

**Abbreviations**
ADE: Antibody‐Dependent Enhancement
ASGR1: Asialoglycoprotein Receptor 1
AXL: AXL Receptor Tyrosine Kinase
bnAbs: Broad Neutralising Antibodies
CD147: Cluster of Differentiation 147
CDR: Complementarity‐Determining Region
CDRH: Complementarity‐Determining Region of Heavy chain
CDRL: Complementarity‐Determining Region of Light chain
COVID‐19: Coronavirus Disease 2019
CoVs: Coronaviruses
DPP4: Dipeptidyl Peptidase 4
E: Envelope Protein
ECDC: European Centre for Disease Prevention and Control
Fab: Fragment Antigen Binding
HCAbs: Heavy‐Chain Antibodies
HCV: Hepatitis C Virus
HIV: Human Immunodeficiency Virus
HR1: Heptad Repeat 1
HR2: Heptad Repeat 2
IAV: Influenza A Virus
IgG: Immunoglobulin G
KREMEN1: Kringle Domain Containing Transmembrane Protein 1
LRRC15: Leucine‐Rich Repeat Containing 15
M: Membrane Protein
MERS‐CoV: Middle East Respiratory Syndrome Coronavirus
N: Nucleocapsid Protein
nAbs: Neutralising Antibodies
NRP: Neuropilin
NTD: N‐terminal Domain
PDB: Protein Data Bank
RBD: Receptor Binding Domain
RBM: Receptor Binding Motif
S: Spike Protein
SARS: Severe Acute Respiratory Syndrome
SARS‐CoV: Severe Acute Respiratory Syndrome Coronavirus
SARS‐CoV‐2: Severe Acute Respiratory Syndrome Coronavirus 2
TMEM106 B: Transmembrane Protein 106B
VH: Variable domain of Heavy Chain
VHH: Variable domain of Heavy‐chain‐only antibodies
VL: Variable domain of Light Chain
WHO: World Health Organisation

## 1 Introduction

The last 2 decades have seen the outbreaks of three lethal zoonotic diseases associated with novel coronaviruses: severe acute respiratory syndrome (SARS) in November 2002, MERS in April 2012, and the recent coronavirus diseases (COVID‐19) in December 2019 [1, 2]. CoVs are single‐stranded RNA viruses with a genome size of 26–32 kb, that encode many proteins that may enable them to adapt to a broad range of environments and facilitate cross‐species transmission [3, 4]. SARS‐related coronaviruses belong to coronavirus lineage B genome whereas MERS is the sixth coronavirus and the first beta coronavirus of lineage C that has been found to infect humans [5]. Even as SARS‐CoV‐2 continues its worldwide spread, MERS‐CoV remains a threat.

MERS was first identified in Saudi Arabia in 2012 [2]. Approximately 953 deaths have been reported due to the infection and related complications in 27 countries since 2012 [6]. More crucially, recent outbreaks of MERS infections and deaths in 2024 (5 cases of infections and 4 deaths) have been reported to the ECDC [7] and WHO [8] by the Kingdom of Saudi Arabia (KSA), raising more concern and need to further understand MERS as a potential pandemic coronavirus. The WHO anticipates that more cases of MERS‐CoV infection will be reported from the Middle East and other regions. Additionally, it is expected that cases will continue to be exported to other countries by individuals who have been exposed to the virus.

MERS‐CoV is the most virulent human pathogenic coronavirus known to date, even though only sporadic infections have been reported in the Middle East since 2016. Most recently, it was warned that the Qatar FIFA World Cup 2022 and camel pageant championships might increase the risk of MERS‐CoV transmission and global spread [9]. In 2015, a similar instance occurred with an individual returning to Seoul from Saudi, resulting in 184 infections with 36 deaths [6]. To date, there is no vaccine or treatment available, even while the case‐fatality rate for MERS‐CoV infection is astonishingly high, approximately 36%, 100 times higher than that for COVID‐19.

Studies have shown that humans are infected by coming into contact with infected dromedary camels [10, 11]. A number of Middle Eastern, African, and South Asian Member States have identified MERS‐CoV in dromedary camels. Even though few cases of human infection have been reported outside of the Middle East, recent studies indicate that zoonotic transmission occurs in Member States with occupational exposure to dromedary camels in a number of Member States in the African continent [6]. Researchers identified near‐complete genome sequences of MERS‐CoV from nasal swabs collected from camels in Saudi Arabia, Oman, and Egypt, revealing high genetic similarity to strains found in humans [12, 13, 14]. In addition, a full‐genome sequence of MERS‐CoV was obtained from a camel in Qatar, which closely matched human isolates. This camel‐derived virus also demonstrated efficient replication in human cells, reinforcing the idea that camels serve as a zoonotic source for MERS‐CoV transmission to humans [15].

A person suffering from MERS typically experiences fever, coughing, shortness of breath and sometimes gastrointestinal symptoms, such as diarrhoea [16]. Occasionally, pneumonia may also occur [6].

MERS‐CoV structural proteins consists of a spike protein (S), envelope protein (E), membrane protein (M), and nucleocapsid protein (N) [17, 18]. As a type I transmembrane glycoprotein, the S protein consists of subunits S1 and S2 and is located on the virus surface as a trimer [17, 18]. The protein plays a pivotal role in binding, fusion, and entry into the host cell. A receptor binding domain is found on the S1 subunit that binds to the dipeptidyl peptidase 4 (DPP4) receptor on the host cell. To allow membrane fusion, the S2 subunit has two heptad repeats, HR1 and HR2, which rearrange to form a six‐helix bundle. The E and M proteins in the membranes play indispensable roles in virus assembly, budding, and intracellular trafficking [17]. Upon entering cells, the MERS‐CoV infects type I and type II alveolar cells through the DPP4. Most of the virus has been detected in respiratory secretions, with lower respiratory tracts having the highest viral loads [19].

Broad neutralising antibodies (bnAbs) have been successfully developed against global circulating viruses such as influenza A virus (IAV), hepatitis C virus (HCV), and human immunodeficiency virus (HIV) [20, 21, 22, 23]. nAbs, therefore, represent an innovative therapeutic or prophylactic strategy against coronaviruses. In most cases, nAbs for SARS‐CoV‐2, SARS‐CoV and MERS‐CoV are directed against the receptor‐binding domains (RBD) of S1 subunit. This prevents viruses from attaching to target cells. However, these RBD antibodies are usually strain specific, which results in the rapid emergence of new variants [24, 25]. Apart from the classical nAbs, another interesting but less‐explored groups of MERS‐CoV antibodies elicited by the S protein are non‐neutralising antibodies (nonnAbs) that are able to offer protection in mice models [26]. These antibodies tend to prevent S protein interaction with sialoglycans on mucins rather than prevent interaction with DPP4.

Analysing the mechanism of action of antibodies against MERS‐CoV is the primary focus of this review. While previous review papers exist for MERS‐CoV and SARS‐CoV/SARS‐CoV‐2 [17, 19, 27, 28], a detailed and updated structure‐function analysis of MERS‐CoV‐antibody interaction and neutralisation is lacking and is crucially needed as coronaviruses become global public health concerns. Here, we provide an update on how the MERS‐CoV evolution and spike protein mutations can lead to a broadly promiscuous receptor usage [4], thwarting efforts towards therapeutics and vaccine development. Besides, we provide a detailed perspective on the immune response and how various nAbs target the MERS‐CoV spike glycoprotein at the molecular level. Ultimately, this review provides insights, which could be applied for the development of broad‐spectrum, potent nAb therapy and next‐generation recombinant vaccine design against CoVs.

## 2 MERS‐CoV Receptor Recognition VS. SARS‐CoV and SARS‐CoV‐2

**FIGURE 1:**
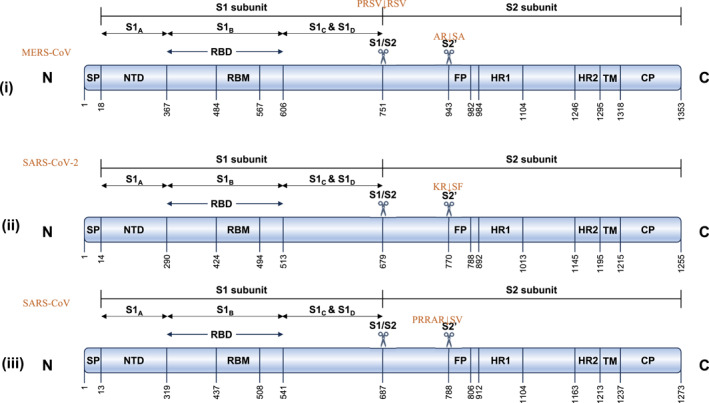
Bar diagrams showing the domain architecture of (i) MERS‐CoV, (ii) SARS‐CoV and (iii) SARS‐CoV‐2 spike glycoprotein. Spike protein is a secretory protein with a short N‐terminal signal peptide (SP). The two domains S1 and S2, which are proteolytically cleaved by host proteases during the spike polyprotein processing are shown on the N‐terminal and C‐terminal respectively with the cleavage site shown as a scissors and shown above the scissors for each spike. A second cleavage site in S2 is further shown. The S1 domain is further divided into four subunits (S1A‐S1D). These four units are divided into two larger units: an N‐terminal domain (NTD: S1A) and a C‐terminal receptor binding domain (RBD) responsible for binding to a protein receptor on the target cell. On the other hand, the S2 subunit begins by adopting a metastable, spring‐loaded configuration, comprising a globular head containing the fusion peptide (FP), heptad repeat 1 and 2 (HR1/2), and a transmembrane domain (TM) and a cytoplasmic tail (CP).

The replication process of coronaviruses including the recent SARS‐CoV‐2, SARS‐CoV and MERS‐CoV follow a very similar pathway except for receptor specificity [17]. Coronaviruses recognise a variety of host receptors with their S protein, which is divided into S1 and S2 subunits [17] (Figure 1). The S1 subunit of the S glycoprotein is further sub‐divided into S1_A,_ S1_B_ (which includes the receptor binding domain, RBD), S1_C_ and S1_D_ domains (Figure 1). The MERS‐CoV RBD recognises the main host receptor dipeptidyl peptidase 4 (DPP4) (expressed in the lungs) [29] and S1_A_ binds sialoglycans [10]. Following attachment of S1, the S2 subunit mediates viral membrane fusion with highly conserved fusion peptide [30]. The fusion peptide is activated through proteolytic cleavage at a site found immediately upstream S2 subunit [31]. After membrane fusion, the viral RNA is released into the cytoplasm for replication and transcription with the help of RNA‐dependent‐RNA‐polymerase (RdRp). Mature virions are then released from the primary cells and continue to infect new target cells [32].

Previous studies have shown the reason behind the faster spread of the COVID‐19 pandemic compared with MERS or SARS [17]. One of the main reasons for this disparity is the structural and amino acid sequence differences in the S proteins among these three coronaviruses. SARS‐CoV‐2 and SARS‐CoV have identical furin‐like S2′ cleavage site at KR↓SF with P1 and P2 basic residues and a P2′ hydrophobic Phe downstream to the internal fusion protein. However, the S glycoprotein of SARS‐CoV‐2 has three extra amino acids upstream to the single Arg↓ cleavage site P1 forming PRRAR↓SV sequence, which is similar to a canonical furin‐like cleavage site often found in highly virulent human influenza viruses [33]. The presence of this furin‐like cleavage site in SARS‐CoV‐2 facilitates the S protein priming and might increase the efficiency of the spread of SARS‐CoV‐2 as compared to other beta coronaviruses. It is imperative to note that in the MERS‐CoV S protein, the corresponding cleavage site P1 sequence is PRSV↓RSV and the S2’ cleavage site is AR↓SA, suggesting a somewhat less favourable cleavage by furin [34]. Consequently, monitoring mutations in MERS‐CoV S1/S2 and S2' cleavage sites can provide an early warning before more virulent emerging CoVs infect humans.

The genome of SARS‐CoV‐2 is 79.6% identical to the genome of SARS‐CoV whereas MERS‐CoV is less genetically related (about 50% identity) [17, 28]. While both SARS‐CoV‐2 and SARS‐CoV virus strains can infect humans with the same ACE2 receptor [35], the SARS‐CoV‐2 S glycoprotein has a 10–20‐fold higher affinity for human ACE2 than does the SARS‐CoV S glycoprotein [36]. There are distinct structural differences between the receptor‐binding domains of SARS‐CoV and SARS‐CoV‐2 S proteins, which represent changes in amino acid sequences that are energetically favourable for the SARS‐CoV‐2 spike to interact more efficiently with the ACE2 receptor.

**FIGURE 2:**
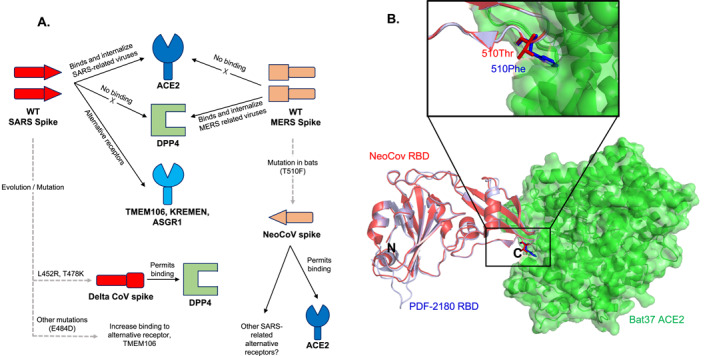
A schematic showing the effect of spike protein mutation on receptor binding and structural analysis of NeoCov‐ACE2 receptor interaction. (A) SARS‐CoV‐2 and SARS‐CoV wild type spike glycoproteins primarily bind ACE‐2 receptor and other alternative receptors like the TMEM106. Similarly, MERS‐CoV WT spike primarily recognises DPP4. Conversely, sarbecoviruses do not recognise DPP4 while merbecoviruses do not recognise ACE‐2 as receptor proteins. Further, a E484D mutation in SARS‐CoV‐2 causes an increased affinity to an alternative receptor, TMEM106. Evolution and mutation of the spike glycoprotein of the SARS‐CoV‐2 virus (giving rise to the Delta variant of concern, VOC) causes the Delta VOC to bind to DPP4 while mutation and evolution of the spike of NeoCov, a bat merbecovirus‐like spike binds ACE‐2. (B) Superposition of NeoCov and PDF‐2180 RBD structures show that a The Thr to Phe mutation in RBD allows NeoCov to bind to and utilise ACE2 as a receptor protein. Superimpose 7WPO with 7WPZ. Mutation of 510Thr to Phe shows the side chain protruding more into the ACE2 binding pocket, explained why it binds stronger. Surprisingly, in the PDB coordinate, the residue does not show any interaction.

**FIGURE 3:**
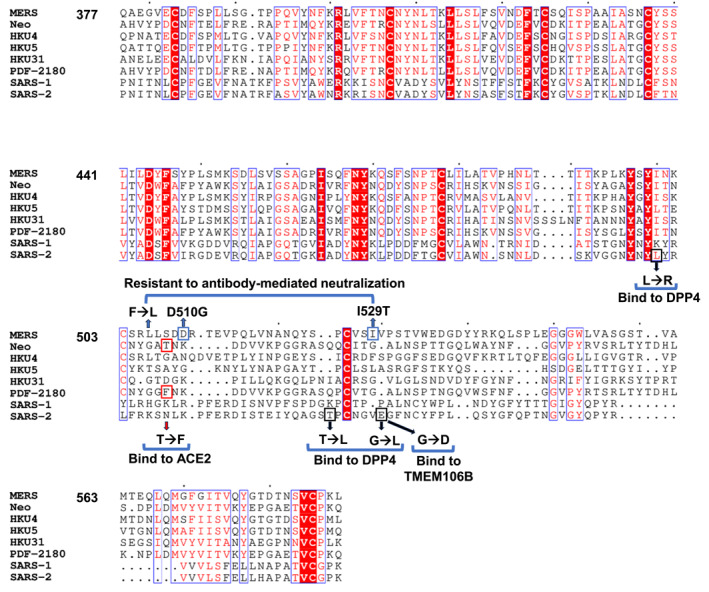
Sequence‐based alignment of coronaviruses receptor binding domain of the spike glycoprotein. The conserved residues are shown in red with white font. Mutations and their impact on antibody neutralisation, receptor binding or virus infectivity are indicated.

As of now DPP4 seem to remain the bonafide receptor for MERS‐CoV [37]. However, recent studies show that SARS‐CoV‐2 and SARS‐CoV viruses may utilise multiple alternative receptor proteins apart from the main receptor ACE2 for cellular internalisation and infection [17]. SARS‐CoV‐2 omicron variant exhibits altered cell tropism and an associated change in the cell entry pathway, indicating that emerging variants may use alternative receptors to escape the immune pressure against ACE2‐dependent viral entry provided by vaccination against RBD [38]. Indeed, previous studies have shown the propensity for cross‐species transmission due to their ability to bind receptors of new hosts [4]. Emerging evidence suggests that the promiscuous receptor usage ability of the SARS‐CoV‐2 virus and its mutants or variants [38, 39, 40, 41] may have driven its faster spread, infectivity and persistence. For example, recent studies have discovered multiple alternative receptors for SARS‐CoV‐2 and or SARS‐CoV such as TMEM106 B [42, 43], CD147 [38, 44], NRP [40, 41], ASGR1, KREMEN1 [38], LRRC15 (in cooperation with ACE2) [39] or AXL [38, 45], with SARS‐CoV‐2 particularly utilising all of these alternative receptors [38] (Figure 2A). In addition, a conserved RGD motif (Arg‐Gly‐Asp, residues 403–405) in SARS‐CoV‐2 may allow it to use integrins as cell receptors [46]. Notably this RGD motif is exclusive to SARS‐CoV‐2 S protein and is not present in other coronaviruses, including MERS‐CoV [46]. SARS‐CoV‐2 S protein interaction with receptors such as the cluster of differentiation 147 (CD147) is linked to unique symptoms of COVID‐19 such as elevated blood glucose levels in infected patients, retarded COVID‐19 risk in women, enhanced susceptibility in geriatrics, and greater infection susceptibility of T cells [47]. It is noteworthy to mention that MERS‐CoV S glycoprotein could not (or may not) recognise or bind to any of these receptor proteins. The obvious differences in the S protein receptor‐binding domain sequences (Figure 3) including differences in glycosylation may be responsible for this discrepancy [48]. However, structural information describing how these alternative receptors engage the SARS‐CoV‐2 and or SARS‐CoV S proteins is necessary to fully understand why MERS‐CoV is or may be unable to utilise these multiple secondary receptor proteins.

### 2.1 Evolution and Mutation of CoV S Protein Permits Receptor Promiscuity and Cross‐Reactivity With Unknown Consequences

Despite the above observations, recent studies [49, 50] have revealed the possibility of MERS‐CoV (merbecoviruses') evolution through genetic recombination with sarbecoviruses, such as SARS‐CoV‐2 and SARS‐CoV, granting it the ability to utilise human ACE2 (hACE2) as an alternative receptor (Figure 2A).Yan and colleagues demonstrated that NeoCoV, a potential MERS‐CoV ancestor in bats, can use bat ACE2 as its entry receptor. Interestingly, the researchers show that NeoCoV S pseudotyped virus, which contains a Thr510Phe mutation in the RBD (Figure 2A and 2B), enters cells expressing hACE2. This finding points to the potential zoonotic threat posed by MERS‐CoV evolution and mutation by gaining the ability to use hACE2 as an entry receptor and, thus, could also coinfect ACE2‐expressing cells with SARS‐CoV‐2. It would be interesting to investigate whether the Thr510Phe mutant NeoCoV S pseudotyped virus can utilise the previously discussed alternate SARS‐CoV‐2 and SARS‐CoV human receptors.

Similarly, while DPP4 is regarded as the receptor for MERS‐CoV, DPP4 was closely associated with elevation of disease severity in comorbidities, especially in type 2 diabetes mellitus during the COVID‐19 pandemic [51]. As such, DPP4 inhibitors were proposed as promising new class of medications that could be used to prevent and treat COVID‐19. To unravel the mechanism by which DPP4 affects COVID‐19, different studies using bioinformatics tools, molecular simulations and protein docking showed high affinity between human DPP4 and the SARS‐CoV‐2 RBD [1, 51](Figures 2A and 3). In particular, comparative binding affinity confers that Delta‐CoV‐2: DPP4 shows close proximity with MERS‐CoV: DPP4. The delta variant exhibits mutations in Leu452Arg and Thr478Lys, actively engaging in DPP4 interaction and strengthening DPP4 binding (Figure 2A). Similarly, the alpha and gamma variants, featuring the Glu484Lys mutation in the S glycoprotein, also demonstrate interaction with DPP4 [51]. Consequently, the mutation‐induced changes, particularly with Leu452Arg, Thr478Lys, and Gly484Lys, render the DPP4 interaction with the spike protein more favourable. Additionally, disturbances in adjacent residues, namely Tyr495, Gln474, and Tyr489, are apparent due to Leu452Arg, Thr478Lys, and Glu484Lys, respectively.

Interestingly, residue at position 484 of S‐glycoprotein seems to be a crucial residue for the SARS‐CoV‐2 engagement with other receptors [42] apart from the well‐known ACE2. A previous study reported that TMEM106B is essential for the infection of several human cell lines that express ACE2 at a low or an undetectable level [43]. Using ACE2‐and TMEM106B knockout NCI‐H1975 cells (ACE2^KO^andTMEM106B^KO^), the researchers demonstrated that TMEM106B can support SARS‐CoV‐2 infection independently of ACE2 [43]. Notably, sequencing analysis uncovered that a change in amino acid from Glu484 to Asp in the S protein (Figures 2A and 3), likely arising during virus passaging, caused heightened infectivity [42].

The identification of the numerous alternative receptor proteins that has endowed the SARS‐CoV‐2 virus with unique capabilities serves as a warning to look out for MERS‐CoV evolution and mutation in the future. Such evolution evokes crucial questions: (1) if the MERS‐CoV spike evolves to utilise SARS‐CoV‐2 receptor proteins, does this evolution pose the threat of MERS‐related pandemic with COVID‐19 symptoms? (2) Will existing MERS‐CoV‐specific antibodies still be effective against new MERS‐CoV infection? (3) Is there a potential threat of multiple coronaviruses pandemics in the future? The recent reemergence of MERS in the Middle East as COVID‐19 continues to wreak havoc makes this particular question urgently important. (4) What kind of therapeutics or vaccines will be effective in the case of such pan‐CoV pandemics scenarios?

MERS‐CoV variants seen during the Korean outbreak in 2015 contain polymorphisms Asp510Gly and Ile529Thr in their RBD, and these variants can be transmitted to other people [52]. In DPP4‐expressing cells, researchers demonstrated robust entry of Asp510Gly and Ile529Thr mutants via the S glycoprotein, and these mutations managed to resist antibody‐mediated neutralisation (Figure 3). It is extremely concerning to know that single point mutations in MERS‐S can make the virus resistant to antibody‐mediated neutralisation. Studies geared towards tracking important sporadic mutations in the MERS‐CoV and other CoV spike and the impact of these mutations on receptor binding, cellular entry, infection, and disease should be of paramount research focus to avoid any future MERS‐related or multi‐CoV‐related pandemics.

## 3 Immune Response and Antibody Elicitation Against MERS‐CoV S Glycoprotein

The primary focus of vaccine design targets the recombinant S protein of coronaviruses, including MERS‐CoV. Vaccine design may target the full‐length S glycoprotein trimer and its subunits (S1, S2), the N‐terminal domain (NTD), the RBD (Figure 1), with or without adjuvants to enhance their immunogenicity [53]. Because of its crucial role in binding to cell surface receptors, the RBD (comprising of the receptor‐binding motif, RBM and the S1_B_CD region) of the S glycoprotein is usually the go‐to domain for antibody response [17, 28, 54, 55, 56]. Studies have shown the potency of both RBD (S1) and non‐RBD (S2)‐induced neutralising antibodies. Furthermore, shorter fragments of the RBD alone such as aa358–588, aa367–588, aa377–588, and aa367–606 [57] have also shown the capacity to elicit neutralising antibodies in vaccine design. Among these RBD fragments, the S377–S588 and its human Fc fusion form (S377‐S588‐Fc) is the most potent fragment that induces the highest immune responses and neutralising antibodies in immunised animals [58]. Notably, the intranasal administration of the RBD domain of the S protein on MERS‐CoV elicits a more robust response from the local mucosal immune system in lung tissue compared to subcutaneous immunisation [59]. Because antibodies that target the RBD domain have a better chance of abrogating S protein‐receptor, DPP4 interaction, this group of antibodies elicit more potent neutralising activity against MERS‐CoV infection than non‐RBD (N‐terminal or S2)‐specific antibodies. However, due to the hypermutation of the RBD, mutant or variant coronaviruses easily evade these group of antibodies as observed with the SARS‐CoV‐2 viral mutants that became resistant to antibody therapeutics [17, 28].

Consequently, new research efforts are constantly being developed to design pan‐coronavirus antibodies that are effective in blocking emerging variants or mutants. Some of these antibodies include the single domain antibodies (also known as variable domains of heavy‐chain antibodies (HCAb) found in Camelidae or VHH antibodies [60, 61, 62]; later described in detail: Section 4.1), which target cryptic regions on the spike trimer and non‐RBD stem helix‐antibodies [63] of the S2 region (later described in detail: Section 5.1) that elicit antibody dependent cellular phagocytosis (ADCP). Because of their ability to neutralise MERS‐CoV in addition to or independent of blocking receptor interaction, these group of antibodies have shown extreme potency against multiple coronaviruses and mutants [17, 28]. Several RBD and non‐RBD‐antibodies discussed in detail later in this review paper have been shown to neutralise MERS‐CoV in in vitro assays as well as offer protection in virus‐challenged mice models [63, 64, 65, 66, 67].

Additionally, the use of prefusion‐stabilised spike protein has been a breakthrough in vaccine and antibody design, particularly for viruses like SARS‐CoV‐2 [68], a technique that has also been adopted in the design of MERS‐CoV spike protein [69]. The spike protein on the virus surface exists in a metastable prefusion state, which is the form recognised by the immune system. By stabilising the protein in this conformation, researchers can create vaccines that elicit strong immune responses, generating antibodies that target the virus before it can infect cells. This approach has been crucial in the development of effective COVID‐19 vaccines, as it enhances the immunogenicity and stability of the spike protein, leading to better protection against infection [70]. Furthermore, prefusion‐stabilised spike proteins are valuable in designing therapeutic antibodies, as they provide a precise target for neutralisation.

## 4 Mechanism of Cross‐Reactivity/Neutralisation of MERS‐CoV RBD‐Specific mAbs

A search for broad‐spectrum antibodies against coronaviruses is a crucial research goal as the emergence of coronaviruses continue to plague the human population. Ideally, discovering and engineering broadly neutralising antibodies (bnAbs) that can cross‐neutralise the three major circulating coronavirus (MERS‐CoV, SARS‐CoV‐2 and SARS‐CoV) and their different strains would be a breakthrough.

**FIGURE 4:**
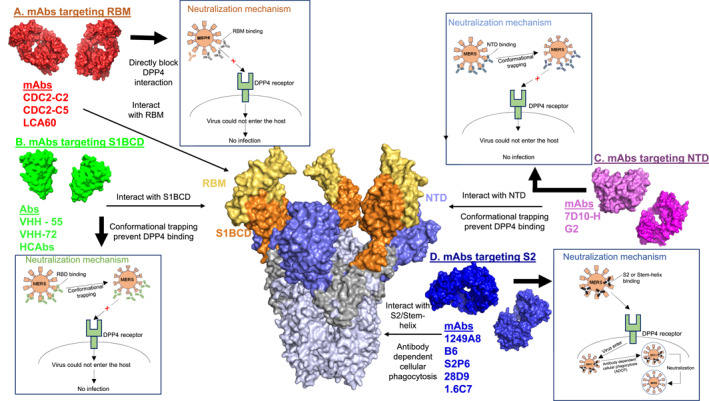
Neutralisation mechanism of the various MERS‐anti‐spike antibodies. There are two groups of RBD‐binding antibodies (RBM and S1BCD). (A) The RBM‐antibodies represent a group of antibodies that bind to the receptor‐binding motif and directly blocks DPP4 binding. (B) S1BCD antibodies bind to regions of the RBD away from the RBM but are still able to block the DPP4 binding either due to conformation trapping or allostery. The S1BCD antibodies are dominated by VHH antibodies. (C) The NTD‐targeting antibodies bind to the N‐terminal domain and may (indirectly) or may not block DPP4 binding. (D) The stem‐helix‐ targeting antibodies are antibodies that bind to the stem helix of the S2 region. While these antibodies do not block receptor binding, they cause antibody‐dependent cellular phagocytosis as a neutralisation mechanism.

In contrast to the current observation with SARS‐CoV and SARS‐CoV‐2 [28], cross‐reactivity and cross‐neutralisation of RBD‐specific (RBM‐ and S1_B_CD‐specific) mAbs among MERS‐CoVs have been significant, showing little disparity from non‐RBD‐specific mAbs, as the critical residues of these domains among MERS‐CoV lineages are well conserved (Table 1). For example, the RBM‐specific bnAb human mAb LCA60, neutralises the England1, MEC/2012, and Jordan‐N3/2012 MERS‐CoV strains [71], and human mAbs CDC2‐C2 (Figure 4A), MERS‐GD27, MCA1, and MERS‐4 neutralise multiple MERS strains in combination with human mAbs MERS‐27, m336, and 5F9. Veesler and colleagues reported that LCA60 bound to MERS CoV S glycoprotein directly competing or impeding the DPP4 receptor binding [71]. Moreover, Raj and colleagues established that complementarity determining region of heavy (H) and light (L) chains (CDRH2, CDRH3, CDRL1, and CDRL3) of mAb LCA60 interact with MERS‐CoV RBD, consisting of protein‐protein and protein‐glycan interactions (PDB ID: 6NB3/B4/B5) [71, 76]. Crystal structures of high‐affinity Fab‐MERS‐CoV RBD complex show human mAbs CDC2‐C2, MERS‐GD27, MCA1, MERS‐4 in coaction with antibodies MERS‐27, m336, and 5F9, bind MERS‐CoV RBD (PDB ID: 4ZS6, 4XAK, 5GMQ) [66, 72, 73, 77] and blocks receptor binding.

**TABLE 1:** MERS‐CoV RBD‐targeting monoclonal antibodies.

mAb name and source	Neutralising activity	Targeted region in S protein	Receptor and mAb competition	Neutralising mechanism	Protective efficacy	PDB/Refs
LCA60 mAb Human MERS‐S‐specific	✓ multiple MERS isolates (England1, EMC/2012, and Jordan‐N3/2012)	MERS‐B domain Mostly targets glycan residues (a.a 166, 236, 487, 531, 541) RBD (a.a 489, 493)	✓ with DPP4 binding to MERS‐S‐protein	Inhibition of MERS‐S‐DPP4 interaction	Effectively protect in both prophylactic and postexposure settings. Protect Ad5/hDPP4 transduced wild‐type/IFNAR‐KO mice from challenging of MERS (EMC2012, London1)	6NB5 6NB4 6NB3 [71]
MERS‐27 m336 MCA1 MERS‐GD27 mAbs Human MERS‐S‐specific	✓ multiple strains of PseuV and live virus MERS infection such as (strain EMC2012)	MERS‐RBD (a.a 506, 510, 535, 539, 540, 542) m336: (a.a 553) MERS‐27: (a.a 535, 539)	Strongly ✓ with DPP4 binding	Inhibition of MERS‐S‐DPP4 interaction	Prophylactically and therapeutically prevent and treat MERS (strain EMC2012) challenged hDPP4‐Tg mice, rabbit, marmosets and susceptible mice	4ZS6 4XAK 5GMQ N/A for MERS‐GD27 [72, 73, 74]
RBD‐23D3 RBD‐25E4 RBD‐40G7 RBD‐14F8 RBD‐43E4 mAbs Mouse MERS‐S‐Without Trans‐membrane Domain (SΔTM)	✓ against 14 different S‐PseuV RBD‐14F8: ✘ 3 PseuV mutants (aa. L506 F/D509 G) RBD‐43E4: ✘KOR/CNUH_SNU/016_06 RBD (I529 T) PseuV	MERS‐S‐RBD Most likely target (a.a 534) Also RBD‐14F8: (a.a. 506, 509, 534) RBD‐43E4: (a.a 534, 529)	✓ with DPP4 binding	Inhibition of MERS‐S‐DPP4 Interaction	N/A However, could be a promising candidate.	N/A [65]
JC57‐11 JC57‐14 mAbs Rhesus macaques MERS‐S‐specific	✓ MERS EMC pseudo‐ particles Higher potency compared to CDC2‐C5 E536 R and D539 R mutants partially resistant to JC57‐14 Mutations in 539 and 542 reduced sensitivity to JC57‐14 JC57‐11 neutralised BtCoV‐422 potently	MERS‐RBD JC57‐14 binds the RBD with W535 at the centre CDC2‐C5 matches well with JC57‐14 mode of binding	Interrupts DPP4 binding	Inhibition of MERS‐S‐DPP4 Interaction JC57‐14: (a.a534, 535, 536, and 539). JC57‐11 inhibition of BtCoV‐422 ‐S‐DPP4 Interaction	N/A	JC57‐14: 6C6X [66]
CDC2‐C2 CDC2‐C5 mAbs Human MERS‐S‐specific	CDC2‐C2: Most potent against MERS EMC. Potency comparable with m336 and REGN3051, most potent Abs thus far. ✓ pseudoparticles of 10 MERS strains. Besides, Bisha1 strain E536 R and D539 R mutants resistant to CDC2‐C5. Mutations at positions 506 and 509 complete Loss of neutralising activity for CDC2‐C2 as with ab F11	CDC2‐C2 binds RBD significantly overlap DPP4 binding interface (a.a.500–515) consistent with m336, and MCA1 Mode of binding including (a.a W535)	Interrupts DPP4 binding CDC2‐C2 was blocked by all mAbs tested, like CDC2‐C5 except F11 JC57‐11 could block all mAbs Tested	Inhibition of MERS‐S‐DPP4 Interaction	CDC2‐C2 protect DPP4‐transgenic mice against lethal MERS‐CoV infection MERS‐CoV (strain EMC/2012).	CDC2‐C2: 6C6Z [66]
MERS VHH‐55 (HCAbs) mAb Llama (camel) MERS‐S specific	✓ MERS England1 S PseuV	MERS‐S‐RBD S1_B_ CD Epitope (a.a 513, 542, 506)	✓ with DPP4 binding	Inhibition of MERS‐S‐DPP4 Interaction Conformational trapping	N/A However, has the potential to serve as useful therapeutics and prophylactic against multiple MERS‐CoV	6WAR [75]
28D9 and 1.6C7	Both Abs neutralise authentic MERS‐CoV; 28D9 inhibits OC43‐S, SARS‐S and SARS2‐S pseudotyped VSV infection	Stem helix of the coronavirus spike protein	✓ with DPP4 binding	Unknown mechanism	Protection against lethal dose of MERS‐CoV in the K18 transgenic mouse model	NA

Abbreviations: ✓, neutralising/competitive; ✘, non‐neutralising/non‐competitive; a.a, amino acid; DPP4, Dipeptidyl Peptidase 4; Fab, Fragment‐antigen binding; HCAb, heavy chain antibody; HDPP4‐Tg mice, human DPP4‐transgenic mice; mAb, monoclonal antibody; MERS, Middle East Respiratory Syndrome‐Coronavirus; MERS‐S, MERS‐CoV Spike protein; N/A, Not Applicable; pAb, polyclonal antibody; PseuV, pseudotyped virus; RBD, Receptor Binding Domain; RBM, Receptor Binding Motif; S, Spike protein; S1_B_CD, S1_B_ Core Domain; scFv, single chain variable fragment.

### 4.1 VHH Antibodies Represent Key Pan‐Coronavirus Antibodies

VHH (also known as nanobody due to their uniquely small size∼15 kDa), are emerging antibodies, making up only the variable region of a heavy chain of a camelid antibody (HCAbs) [60, 78, 79]. Contrary to conventional IgG antibodies with both heavy and light chains (*V*_*H*_ and *V*_*L*_), VHHs contain single variable domain and are biophysically advantageous due to their small size, high antigenic affinity, extreme thermal and chemo‐stability, and the ease of large‐scale production [60, 78, 79]. These properties have presented VHH antibodies as robust therapeutic molecules against respiratory pathogens, such as respiratory syncytial virus and coronaviruses and, therefore, made waves of hope for the COVID‐19 pandemic. Due to their small molecular weight property and long CDR3s, VHH antibodies can target naturally inaccessible cryptic epitope regions of the spike glycoprotein (specifically the S1_B_CD region) (Figure 4B), which enables them to exhibit picomolar affinities and broad [60, 62, 77]cross‐reactive and neutralising activity against different strains of SARS‐CoV or MERS‐CoV as exemplified by SARS VHH‐72 and MERS VHH‐55 [80]. These antibodies cross‐react and cross‐neutralise multiple MERS strains through a combination of receptor interaction inhibition and conformational trapping. For example, the VHH antibodies (VHH‐1, VHH‐4, VHH‐83, and VHH‐101) and their engineered HCAb forms, (HCAb‐1, HCAb‐4, HCAb‐83, and HCAb‐101) exhibited highly potent and broad cross‐reactivity and neutralisation against multiple MERS‐CoV isolates [60]. Notably, the epitopes targeted by these antibodies map to common hotspot residues (Asp539, Glu536, Ile529, Val534, Glu536) consistent with some of the residues (Asp537, Asp539, Tyr540, and Arg542) necessary for DPP4 interaction.

It has been shown that most nAbs target S1_B_CD of the RBD [64]. In vitro neutralisation assays tested against MERS‐CoV England1 S pseudovirus showed that S1_B_CD‐antibodies MERS VHH‐55, −12, −34 and −40 have a high‐affinity and neutralises the virus with IC_50_ ranging between 0.9 and 193.3 nM [75]. The crystal structure of mAb MERS VHH‐55 bound to MERS‐CoV RBD show many interactions between the CDRs of the mAbs MERS VHH‐55 and MERS‐CoV RBD (PDB ID: 6WAR) [75]. Aligning the structures of receptor DPP4:MERS‐CoV RBD complex with mAb MERS VHH‐55:MERS‐CoV RBD complex showed that the mAb MERS VHH‐55 CDR3 is looped on top of the receptor DPP4 binding interface, impeding DPP4 binding to the MERS‐CoV RBD [75]. Other than obstructing DPP4 engagement, conformational trapping is also a likelihood, as MERS‐CoV RBD shows multiple conformations that mediate the opening of the RBMs, and in turn the possibility to bind to host cells [71, 75]. Moreover, the structure suggests that the residue Arg542 of RBD plays a crucial role in binding to mAb MERS VHH‐55.

Like CDC2‐C5 and CDC2‐C2 mAbs, none of the four VHH antibodies (MERS VHH‐55, ‐12, −34 and −40) could bind to a Asp539Asn variant. VHHs, while quite potent, acquire even higher potency and protective efficacy when engineered into human chimeric HCAbs [60, 80]. This humanisation process of VHHs to make them into HCAbs significantly reduces the immunogenicity risks and improves their overall efficacy. For example, in an experiment with two VHH variant antibodies (VHH‐83 and HCAb‐83), while mice that received the non‐engineered VHH‐83 did not survive, all mice that received HCAb‐83 survived a lethal dose of (10^5^ TCID_50_: median tissue culture infectious dose) MERS‐CoV EMC isolates [60].

### 4.2 Limitations of MERS‐CoV RBD‐Specific Antibodies: Key Amino Acids That Dictate Reactivity and Neutralisation

Functional studies have shown that RBD‐specific antibodies that directly or indirectly block DPP4 binding to the S glycoprotein are relatively safer and more potent than non‐RBD targeting antibodies [81, 82]. However, although RBD‐specific mAbs can neutralise viruses with higher potency, there is a potential risk for viruses to escape neutralisation under selection pressure especially if a single residue epitope is targeted [66]. For example, position 506 of the S glycoprotein is highly variable among MERS‐CoV strains [83, 84] (Figure 3) and can lead to reduced reactivity; this is observed with Phe506Leu S glycoprotein strains reduced binding to the S1_B_CD mAb MERS VHH‐55. Likewise, mAb RBD‐43E4 fails to cross‐react or cross‐neutralise the MERS‐CoV KOR/CNUH_SNU/016_06 strain because of a single Ile529Thr mutation (Figure 3) [66].

Similarly, one of the most critical single‐residues targeted by MERS‐CoV mAbs is Trp535 (Table 1). The RBD‐specific mAbs D12, 4C2, MERS‐27, and JC57‐14 all mainly neutralise MERS‐CoV with interaction centred around Trp535 [74] (Figure 3). Other critical residues include Leu506, Asp509, Asp510, Arg511, Val534, Ile529, Glu536, and Asp539 [85]. A highly potent neutralising mouse mAb RBD‐14F8 which showed broad activity and neutralisation against 14 different MERS s‐pseudoviruses could not cross‐react or cross‐neutralise three pseudoviruses with amino acid mutations Leu506Phe or Asp509Gly [65]. Moreover, four highly potent mAbs (RBD‐23D3, RBD‐25E4, RBD‐40G7, RBD‐43E4) including mAb RBD‐14F8 which equally neutralised all the 14 different MERS s‐pseudoviruses could not cross‐react or cross‐neutralise pseudoviruses with amino acid mutations (Val534Ala) [65]. Similarly, double mutants (Glu536Arg, Asp539Arg) and (Leu506Phe, Asp509Gly) completely abrogated neutralising activity by human mAbs CDC2‐C5 and CDC2‐C2, respectively [66] and a single substitution (Arg511Ala) completely abolished RBD‐Fc binding to mAb Mersmab1 [85]. These single or double escape mutant scenario seems to be rampant with RBM‐specific antibodies possibly due to the high selection pressure of the RBD of the virus to undergo mutations for better adaptation to broad and efficient host infection.

## 5 Mechanism of Cross‐Reactivity/Neutralisation of MERS‐CoV Non‐RBD‐Specific mAbs

Due to their relatively poor neutralising activities, non‐RBD MERS‐CoV mAbs have not been well characterised except for few including the mAb‐7D10 and mAb‐G2 (Figure 4C), which potently neutralise multiple MERS‐CoV strains [67, 86] (Table 2). The mAbs 7D10 and G2, similar to MERS VHH‐55 also exhibit the unique property of binding to the S1‐non‐RBM (S1‐NTD) epitope (Figure 4C) and yet interfering with DPP4 binding, thereby potently inhibiting multiple MERS‐CoV isolates. Overall, the broad reactivity and neutralising activity of these groups of antibodies lies in their ability to recognise different epitopes of the S glycoprotein and engage more than one neutralising mechanism. G2 mAb can neutralise a broad range of MERS‐CoV pseudoviruses by interacting with conserved residues including Lys27, Ser191, Asn193, Ala197, and Asn199, among all 232 homologue sequences of the S glycoprotein aligned [67, 86]. Notably, a few escape mutants exist against G2 with S glycoprotein amino acid substitutions Ser28Pro or Gly198Asp. These mutations exist naturally in the S glycoproteins of two different MERS‐CoV strains (camel/UAE_B42 and Riyadh_2014KSA_349, respectively). The natural occurrence of Ser28Pro and Gly198Asp mutations may indicate that like RBD‐specific mAbs, non‐RBD‐specific mAbs may equally be under selective pressure. This implies that the ideal antibody therapeutics against coronaviruses is probably a combination of two or more of RBM‐specific nAbs, S1_B_CD‐specific nAbs and the NTD and S2 specific nAbs.

**TABLE 2:** MERS‐CoV Non‐RBD‐targeting monoclonal antibodies.

mAb name and source	Neutralising activity	Targeted region in S protein	Receptor and mAb competition	Neutralising mechanism	Protective efficacy	PDB/Refs
JC57‐13 FIB‐H1 CDC2‐A2 CDC2‐A10 mAbs Rhesus macaques Human MERS‐S‐specific	Reaching no more than 90% neutralisation	Non‐RBD S1 domains	Unlikely to interrupt MERS‐S DPP4 receptor binding	Unknown mechanism	N/A	N/A [66]
7D10 mAb Mouse MERS‐S specific	Inhibits cell entry of spike protein with high potency ✓ the infectivity of PseuV and live MERS	S1‐NTD (aa. 18–353) Critical residues: (a.a 24, 26, 188, 235, and 222‐linked)	7D10‐H interfere DPP4 binding to S trimer	Inhibition of MERS‐S‐DPP4 Interaction and the pre‐fusion to post‐fusion conformational change of the S protein.	N/A However, has the potential to serve as useful therapeutics and prophylactic against multiple MERS‐CoVS.	6J11 [86]
G2 mAb Mouse MERS‐S specific	✓ broadly against an array of MERS strains	S1‐NTD conserved residues (a.a 27, 191, 193, 197, 199)	✓ with DPP4 binding	Strong inhibition of MERS‐S‐DPP4 Interaction	Confer protection against lethal challenge in animal models	6PXG [87]
1249A8	✓ neutralises (SARS‐CoV‐2) original strain, delta, and omicron VoC, (SARS‐CoV), (MERS‐CoV)	S2 residues 1131–1171 Targets the stem helix (SH) region of CoV spike (S) protein (residues 1148–1158).	✓ mAbs CV3‐25, CC40.8, and SP26	Antibody dependent cellular phagocytosis Also, disrupts the secondary structure and refolding events required for CoV post‐fusion	Demonstrated significant prophylactic activity in K18 hACE2 mice infected with SARS‐CoV‐2 Protected SARS‐CoV‐infected hamsters from weight loss	8FAX [63, 67, 88, 89]
76E1	✓ neutralises two *α*‐coronaviruses (HCoV‐229E and HCoV‐NL63) and five *β*‐coronaviruses (SARS‐CoV‐2, SARS‐CoV, MERS‐CoV, HCoV‐OC43 and HCoV‐HKU1)	S2 peptide 809–833. Main S2 residues involved in the interaction are Arg815, Glu819 and Phe823	✓ 76E1 targets the S2’ site and the fusion peptide	Inhibit S2’ cleavage, thus avoiding S2’ fragment refolding and blocks the virus–host cell membrane fusion	Prophylactically and therapeutically prevent and treat SARS‐CoV‐2 and HCoV‐OC43 in mice. Reduced lung viral titres observed in 76E1 treated mice	7X9E [90]
CC25.106, CC68.109, and CC99.103	✓ neutralises SARSCoV‐2, SARS‐CoV, and MERS‐CoV	S2 stem helix of the coronavirus spike protein	✓ ACE2 and DDP4 receptor interaction	Could potentially block the fusion process of the virus with the host cells	Protection against diverse beta coronaviruses observed in mice	8DUG, 8DGV, 8DGX [89]

Abbreviations: ✓, neutralising/competitive; ✘, non‐neutralising/non‐competitive; a.a, amino acid; DPP4, Dipeptidyl Peptidase 4; Fab, Fragment‐antigen binding; HCAb, heavy chain antibody; HDPP4‐Tg mice, human DPP4‐transgenic mice; mAb, monoclonal antibody; MERS, Middle East Respiratory Syndrome‐Coronavirus; MERS‐S, MERS‐CoV Spike protein; NTD, N‐Terminal Domain; pAb, polyclonal antibody; PseuV, pseudotyped virus; RBD, Receptor Binding Domain; S, Spike protein; scFv, single chain variable fragment.

A recent study has shown that antibody 76E1 neutralises two *α*‐coronaviruses (HCoV‐229E and HCoV‐NL63) and five *β*‐coronaviruses (SARS‐CoV‐2, SARS‐CoV, MERS‐CoV, HCoV‐OC43 and HCoV‐HKU1) [90]. This antibody targets the S2' site and the fusion peptide, which prevents the cleavage of S2' and blocks the fusion between the virus and host cell membrane. 76E1 interacts mainly with Arg815, Glu819, and Phe823 in the S2 domain, and these residues are highly conserved among the four coronavirus genera. It is noteworthy that Arg815 is the S2' cleavage site, which initiates fusion activation after cleavage [91]. Since 76E1 showed good binding to peptides 809–833 from *α*‐, *β*‐, *γ*‐ and *δ*‐coronaviruses, it may have therapeutic applications against these viruses, including MERS‐CoV and S2’ site represents a promising target for broad coronavirus inhibitors.

### 5.1 S2‐Targeting CoV Stem‐Helix Antibodies

Another group of non‐RBD‐specific broadly neutralising antibodies is the stem‐helix antibodies (Figure 4D). These bnAbs such as 1249A8 [63], B6 [50], S2P6 [88], 28D9 and 1.6C7 [84] target a conserved stem helix epitope (residues 1148–1158) in the S2 subunit of SARS‐CoV‐2 spike protein. As potent bnAbs, they are also effective against SARS‐CoV, MERS‐CoV and or Neo‐CoV infections, suggesting that antigens containing the stem helix region may be used for the development of pan‐β‐CoV vaccines. Indeed, 32 bnAbs targeting the conserved S2 stem helix region of the betacoronavirus spike fusion machinery were isolated from SARS‐CoV‐2 recovered‐vaccinated donors [89]. Three of these stem‐helix bnAbs CC25.106, CC68.109, and CC99.103 were found to protect against deadly human betacoronaviruses, SARS‐CoV, SARS‐CoV‐2, and MERS‐CoV in aged mice. The therapeutic potential of these bnAbs in humans needs to be assessed in future studies.

## 6 MERS Non‐Neutralising Antibodies

**FIGURE 5:**
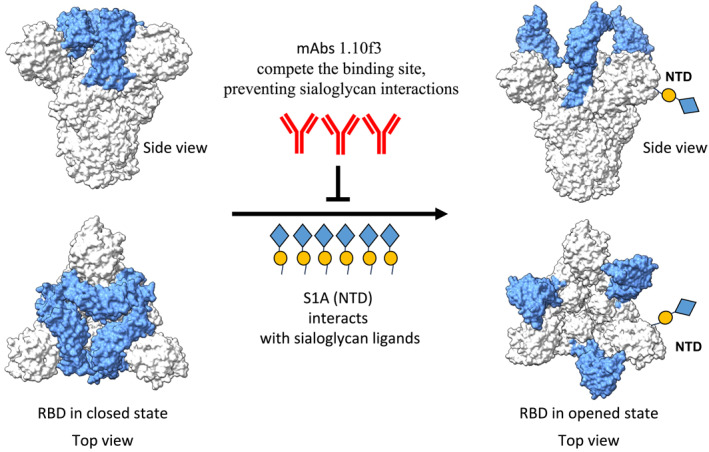
Protective mechanism of MERS‐CoV non‐neutralising antibodies. The closed and open confirmation as well as the top and side view of the spike glycoprotein structure is shown. The binding of non‐neutralising mAb 1.10f3 to the MERS‐S1_A_ or N‐terminal domain (coloured in blue) inhibits the interaction of MERS‐CoV S glycoprotein with the sialoglycans (shown as blure square heads and yellow circle tails) on mucins, in turn inhibiting the sialoglycan‐mediated human MERS‐CoV infections.

Another interesting group of antibodies with unexpected benefits is MERS non‐neutralising antibodies (non‐nAbs) (Figure 5). Besides their property of not being able to neutralise, non‐nAbs may also be associated with unintended negative outcomes known as antibody‐dependent enhancement of diseases (ADE) [83, 92, 93]. Contrary to the general ADE concern of non‐nAbs [93, 94, 95], some positive outcomes have been observed with MERS non‐nAbs. An example of a non‐nAb is the mAb 1.10f3 that targets the sialic acid‐binding domain (confirmed by domain‐level epitope mapping) of MERS S protein [96, 97]. The binding competition assays suggested the binding of mAb 1.10f3 on the S1_A_ domain [96]. The binding of non‐neutralising mAb 1.10f3 to the MERS‐S1_A_ domain inhibits the interaction of MERS‐CoV S protein with the sialoglycans on mucins, in turn inhibiting the sialoglycan reliant human MERS‐CoV infections [96]. Further investigation is needed to have a better understanding of the efficacy of non‐nAbs compared to nAbs, especially in terms of protection in animal models and human clinical trials.

## 7 Concluding Remarks

1. The emergence of the COVID‐19 pandemic shifted our attention from other pathogens including other coronaviruses like MERS. However, the continues outbreaks of coronaviruses, especially MERS, which re‐emerged in 2024 amidst the COVID‐19 pandemic means that we must continue to treat all coronaviruses as potential pandemic threats.
2. The high sequence variability of viral glycoproteins poses an enormous obstacle to the development of vaccines or therapies based on mAbs. However, broadly neutralising mAbs have been discovered against viruses known to undergo extreme antigenic drift such as HIV and influenza viruses. Since several highly pathogenic coronaviruses have recently emerged, this warrants the importance of developing vaccines and therapeutics to protect humans against a variety of coronaviruses, including MERS‐CoV.
3. Here, we have discussed how the evolution of coronaviruses may dictate receptor engagement, transmission, and infection and affect antibody therapeutics and vaccine development. Particularly, we pointed out that the continuous mutation and evolution of coronaviruses is driving the propensity of these viruses to utilise multiple host receptor proteins allowing for cross‐species transmission and infection. Using the SARS‐CoV‐2 spike mutation as an example, which gave rise to many variants of concern, we discussed why similar emerging mutations and evolutions of MERS‐CoV and other CoVs could lead to unknown consequences and, perhaps, a future MERS‐CoV‐related or multiple CoV pandemic scenario.
4. The second aspect of this review focused on MERS‐CoV S protein interaction and neutralisation by antibodies. We discussed in detail the mechanism of cross‐reactivity and neutralisation of nAbs against RBD‐ and non‐RBD epitopes of MERS‐CoV. Our analysis suggests that although RBM‐specific mAbs can neutralise virus with higher potency due to their ability to directly abrogate receptor‐binding, there is a potential risk for viruses to escape neutralisation under selection pressure especially if a single residue epitope is targeted.
5. Further, this review also focuses on the application of single domain antibodies, also referred to as VHHs and stem‐helix‐targeting antibodies, which can target conserved epitopes. These group of antibodies have the unique property of neutralising both MERS and SARS‐related viruses and should be given further attention. BnAbs that target different epitopes of MERS‐CoV as well as could cross‐react and cross‐neutralise other coronaviruses such as SARS‐CoV‐2 and SARS‐CoV might be the holy grail against MERS‐CoV infection. VHH‐HCAb and the stem helix epitope‐targeting antibodies, if explored extensively, has the potential of pan‐coronavirus antibody prophylactics and therapeutics that can overcome viral evolutions and mutations. These antibodies are able to employ multiple neutralisation mechanisms, including direct or indirect blocking of receptor binding, conformational trapping of the spike trimer, and antibody‐dependent cellular phagocytosis.
6. Unfortunately, the low S‐protein sequence identity among the different CoV types explained why the MERS‐CoV‐specific antibodies were ineffective against SARS‐CoV or SARS‐CoV‐2. Thus, it stands to reason that an ideal treatment regimen against these viruses needs to be tailored or specific while research towards discovering more potent pan‐protective antibodies continues. Notably a proof‐of‐concept for these group of pan‐coronavirus antibodies such as the 1249A8, B6 and S2P6 antibodies that target a conserved stem helix epitope of multiple coronaviruses have started to emerge with much potential to be explored. Thus, for broad coronavirus neutralisation, it is appealing to target the S2 fusion machinery, since it contains several significant antigenic sites and is more conserved than the S1 subunit.
7. Furthermore, new mAbs such as SARS VHH‐72, MERS VHH‐55, 7D10 and G2 targeting other functional regions of the MERS‐CoV, SARS‐CoV and SARS‐CoV‐2 S glycoprotein and/or neutralising by different mechanisms are important for developing effective antibody therapies that exhibit broader and more potent neutralising activity against multiple coronavirus strains. In addition, as seen during the COVID‐19 pandemic, cocktail antibodies consisting of two or more mAbs (preferably RBD and non‐RBD‐targeting antibodies) may be more effective against MERS‐CoV. For example, combining VHH antibodies with the stem‐helix antibodies and or N‐terminal‐specific mAbs might offer better protection than the individual antibodies.
8. Moving forward, improving the design of prefusion‐stabilised MERS‐CoV and other coronavirus' spike protein (such as improved hexa‐pro) [68, 70] will aid in the structure‐guided vaccine and antibodies design and development against infection and pandemics.
9. Overall, the analysis that we have conducted could provide crucial insights into how antibodies cross‐react and cross‐neutralise different coronaviruses, which could lead to the development of pan‐CoV vaccines in the future.

## Author Contributions

Conceptualisation, Funding acquisition, Resources: J.S. Writing original manuscript: E.G., Y.K.C., S.N., H.S. Writing – Review and Editing: E.G., S.N., Y.K.C., H.S., E.S.Y., J.S.

## Conflicts of Interest

The authors declare no conflicts of interest.

## Data Availability

The data that support the findings of this study are available on request from the corresponding author. The data are not publicly available due to privacy or ethical restrictions.
